# Verbenone prevents cyclophosphamide-induced oxidative stress, inflammation, fibrosis, and cellular changes in the kidneys of Swiss albino mice by targeting NF-ĸB/TGF-β signaling pathways

**DOI:** 10.22038/ijbms.2025.83541.18091

**Published:** 2025

**Authors:** Mohd Wasim, Syed Mansoor Ali, Syed Ehtaishamul Haque

**Affiliations:** 1 Department of Pharmacology, School of Pharmaceutical Education and Research (SPER), Jamia Hamdard, New Delhi–110062, India; 2 Department of Biotechnology, Jamia Millia Islamia, New Delhi-110025, India

**Keywords:** Cyclophosphamide, Inflammation, Nephrotoxicity, NF-kB signaling, Oxidative stress, Renal fibrosis, TGF-β1, Verbenone

## Abstract

**Objective(s):**

Verbenone was assessed for its protective effect in cyclophosphamide-induced nephrotoxicity in Swiss albino mice.

**Materials and Methods:**

Mice were divided into six groups (n = 6). Vehicle Control, CP 200, VRB 200 + CP, VRB 300 + CP, FF 80 + CP, and VRB 300 *per se*. VRB and FF were given orally for 14 days, while CP was given intraperitoneally only on the seventh day. Mice were sacrificed on the 15^th^ day, and various parameters were examined to ascertain the injury, inflammation, fibrosis, and histological variations in the kidney.

**Results:**

Our results revealed that malondialdehyde, TNF-α, interleukin-6, and IL-1β levels were increased by 248%, 128.5%, 170.68%, and 252%, respectively, and IL-10, catalase, glutathione, and SOD were decreased by 75.75%, 73.58%, 77%, and 81%, respectively in the CP treated group as compared to the control. VRB 300 and FF 80, however, reversed these parameters to normal. In the case of the test drug (VRB 300), the level of malondialdehyde, TNF-α, interleukin-6, and IL-1β levels were considerably decreased by 48%, 41%, 44.5%, and 22.7%, respectively. The levels of IL-10, catalase, glutathione, and SOD were increased by 169%, 228.5%, 208%, and 237%, respectively, as compared to the CP group. VRB 300 and FF 80 also caused a drop in serum creatinine, Uric acid, BUN, and urea levels when compared with the CP group. It reversed fibrosis and cellular architecture to normal, as established by histopathology and immunohistochemistry.

**Conclusion:**

The data confirmed that verbenone significantly plays a role in protection against cyclophosphamide-induced renal damage.

## Introduction

Cyclophosphamide (CP) is considered an important medication for treating solid-tumor cancers and blood malignancies. Besides its use in cancer treatment and as an immunosuppressant, it has been found to help treat nephrotic syndrome (1). As for pharmacokinetics, the metabolism of this drug takes place in hepatocytes. Metabolites of CP, like acrolein and phosphoramide mustard, are formed by a reaction with CYP34A in the liver (2). Phosphoramide mustard can alter DNA replication by attaching to the N7 terminal position of guanine residues and acting as an active metabolite with a strong anticancer impact (2). Acrolein, a short-chain carbon compound that is hydrophobic in nature, is primarily known for its toxic effects rather than any therapeutic benefits. Acrolein is chiefly responsible for the side effects associated with CP, particularly hemorrhagic cystitis, without any anticancer activity (3). Thus, while it has been shown to be effective against cancer cells, CP must be cautiously utilized in treatment or keenly monitored for therapeutic purposes (4). Although CP is often prescribed, its frequent use limits the drug’s usefulness, as it negatively impacts the quality of the patient’s life. Nephrotoxic side effects are among the most common complications caused by its therapeutic doses (5). The kidney is a vital organ that performs crucial intracellular and extracellular activities. The kidney maintains normal body homeostasis by keeping the ionic gradient (pH) and fluid balance. Therefore, a change in the kidney’s normal functioning can severely affect the body’s normal physiology (6). The pathological mechanisms of nephrotoxicity are driven by sustained production of reactive oxygen and reactive nitrogen species (ROS & RNS), reduction in anti-oxidant enzymes, increase in lipid peroxidation (TBARS), and apoptosis (marked by elevated cytochrome c activity and cleaved caspase-3 expression) (5). Moreover, CP also induces inflammation and fibrosis via Nuclear factor erythroid 2- related factor 2 (Nrf 2), NOD-like receptor with pyrine 3 domain (NLRP 3), Transforming growth factor- β1 (TGF-β1), Nuclear factor kappa activated B cells (NF-κB), and p38 activated Mitogen protein kinase (p38 MAPK) pathways (7, 8). These findings emphasize that acrolein-induced oxidative stress, inflammation, and fibrosis are key factors contributing to renal dysfunction. 

At present, there is no standardized therapeutic method to address CP-induced renal toxicity. However, introducing a molecule that can reduce toxicity when given in conjunction with CP may provide an effective solution for managing renal toxicity. Interestingly, researchers are intensively investigating natural products for their pharmacological properties, targeting safety, efficacy, and cost-effectiveness (6). The repurposing of already proven safe and bioactive molecules becomes a preferable target for the research. In this regard, we have chosen Verbenone (VRB) to assess its potential for alleviating CP-induced kidney toxicity as it is a Generally Recognized as Safe (GRAS)” compound by both the FDA and WHO (9, 10) and has proven effective in different indications. VRB is a bicyclic monoterpene that carries a ketone group, a four-membered ring with a carbon-carbon double bond, and is naturally found as one of the key elements of *Rosemarinus officinallis*, *Verbena triphylla L., Chrysanthemum morifolium Ramat, Eucalyptus globulus L., and Piper aleyreanum* (11–13). Some bark beetles release trace amounts of VRB, which comes from the host tree’s antecedent alpha-pinene and functions as an anti-aggregation pheromone (14). The compound VRB exhibits many biological activities, including anticancer (15, 16), antiviral (17, 18), anti-oxidant (19), insecticidal (20, 21), antifungal (22, 23), anticonvulsive (24), and herbicidal (25, 26). However, the nephroprotective potential of VRB has not been investigated so far. Hence, we examined the potential role of VRB on CP-induced kidney damage in an *in vivo* model by measuring changes in oxidative stress, inflammation, and fibrosis via the NFκB and TGF-β pathway. Although terpenes at high doses are typically considered genotoxic, VRB stands out for its relative safety, with an LD_50_ of 3400 mg/kg (p.o.) in mice (27). Based on these findings, we hypothesized that VRB could be a viable therapeutic option for mitigating CP-induced nephrotoxicity. 

## Materials and Methods

### Drugs, chemicals, and reagents used

Endoxan™ - N (cyclophosphamide), Batch No BUY1026, was procured from Baxter Oncology GmbH, Frankfurt, Germany, to induce animal kidney toxicity. The verbenone (CAS no. 1196-01-6) utilized in the study was obtained from TCI Chemicals, India. For immunohistochemistry, antibodies were procured from Santa Cruz Biotechnology. The rest of the reagents and chemicals required for the experiments were of analytical grade. 

### Experimental animals for in vivo study

The* in vivo* investigation employed Swiss Albino mice weighing between 30 and 35 grams. This work was approved by the CPSCEA (protocol No. IAEC/JH/1775), and animals were provided by CAHF, Jamia Hamdard. Before the beginning of the experiment, the animals underwent a week of acclimatization and were housed in a propylene cage with a standard diet, temperature, and humidity. 

### Treatment protocol

The mice were divided into six groups (n=6) and treated, as shown in Table 1. The dose of CP 200 mg/kg IP was given to mice once on the 7^th^ day, and VRB 200, 300 mg/kg p.o., and 80 mg/kg p.o. dose of FF was administered for fourteen days (24, 28, 29). The weights of the mice were taken, and then they were euthanized a day after the final treatment. Blood was withdrawn, and kidneys were weighed and harvested for analysis. In a 10% formalin solution, a segment of kidney tissue was kept for histopathological studies, and the remaining section was preserved at – 80 °C for biochemical estimations (30). 

### Estimation of oxidative stress markers

*Estimation of lipid peroxidation*

The level of malondialdehyde (MDA) was determined to estimate the extent of lipid peroxidation (LPO) by mixing the tissue homogenate with trichloroacetic acid (TCA) and thiobarbituric acid (TBA). Absorbance was measured at 540 nm using the blank as a reference (31). 

*Estimation of superoxide dismutase*

Based on the inhibition of auto peroxidation of pyrogallol, the activity of superoxide dismutase (SOD) was estimated using tissue homogenate. The tissue homogenate was gently combined with Tris-HCl buffer (pH 8.5), followed by the addition of pyrogallol. Absorbance was measured at a wavelength of 420 nm (32). 

*Estimation of catalase*

The rate of disappearance of H2O2 and the level of CAT were measured. The tissue homogenate supernatant was mixed with an H_2_O_2_ solution prepared in a potassium phosphate buffer. Absorbance was measured at 240 nm over three minutes with an interval of one minute (33). 

*Estimation of reduced glutathione*

The level of reduced GSH was estimated by mixing the tissue supernatant with TCA and DTNB, and absorbance was measured at a wavelength of 410 nm (34). 

### Estimation of indicators of kidney function

Twenty-four hours after the final treatment, the mice were euthanized, and blood samples were collected. The samples were then centrifuged at 3500 rpm to separate the serum, which was subsequently stored at -20 °C until analysis. Levels of serum creatinine, uric acid, urea, and blood urea nitrogen were measured by an autoanalyzer (Erba Chem 5x Biochemistry Analyzer, Semi-Automatic) in compliance with the manufacturer’s instructions. 

### Assessment of inflammation

*Assessment of interleukin-6 (IL 6)*

IL-6 levels were assessed using an ELISA kit from Krishgen Biosystems, Mumbai, India. The wash buffer and standard were prepared, followed by the addition of standard (100 μl) and test samples to the wells. The plate was sealed and incubated at 37 °C for 120 min, then washed and blotted. Next, 100 μl of diluted detection antibody was added, incubated for 60 min, and washed again. Avidin-HRP (100 μl) was then added, incubated for 30 min, and washed. Afterward, 100 μl of TMB substrate was added and incubated in the dark for 20 min, followed by the addition of stop solution. Absorbance was taken at 450 nm. 

*Assessment of interleukin-10 (IL 10)*

IL-10 levels were measured using a sandwich ELISA kit from Krishgen Biosystems, India. Reagents were maintained at room temperature, and the wash buffer and standards were prepared according to the protocol. Standard (50 μl) was added to the standard wells, while 40 μl of samples and 10 μl of biotinylated IL-10 antibody were added to the sample wells. Streptavidin-HRP (50 μl) was then introduced to all wells. The plate was sealed, incubated at 37 °C for 60 min, and washed four times. Subsequently, 50 μl each of Substrate A and Substrate B were added, followed by a 10-minute incubation at 37 °C. Finally, 50 μl of stop solution was added, and absorbance was taken at 450 nm. 

*Assessment of interleukin-1β (IL-1β)*

IL-1β levels were assessed using an ELISA kit from Krishgen Biosystems, India. Reagents were maintained at room temperature, and the wash buffer and standards were prepared according to the protocol. Standards and samples (100 μl) were added to the wells, and the plate was sealed and incubated at room temperature for 2 hours. After four washes, 100 μl of diluted detection antibody was added and incubated at room temperature for an hour. Another wash was performed, followed by the addition of 100 μl of diluted Streptavidin-HRP, and the plate was incubated at room temperature for 30 min. The plate was rewashed, 100 μl of TMB substrate was added, incubated in the dark for 15 min, and then stopped with 100 μl of stop solution. Absorbance was taken at 450 nm. 

*Assessment of tumor necrosis factor α (TNF-α)*

TNF-α levels were measured using a sandwich ELISA kit from Krishgen Biosystems. Reagents were maintained at room temperature, and the wash buffer and standards were prepared as per protocol. A 50 μl standard was added to the standard wells, while 40 μl of samples and 10 μl of biotinylated TNF-α antibody were added to the sample wells. Streptavidin-HRP (50 μl) was then added to all wells, and the plate was sealed and incubated for one hour at room temperature. Following incubation, the plate was washed four times with wash buffer and blotted dry. Then, 50 μl each of Substrate A and Substrate B were added, and the plate was incubated for ten minutes at room temperature. Finally, 50 μl of stop solution was added, and absorbance was taken at 450 nm. 

### Routine histopathological analysis (H&E staining)

Histopathological examination was done on kidney samples stored in 10% formalin. H&E staining was used to analyze the cellular architecture of the kidney. The kidney tissue was submerged in paraffin and sliced into thin sections (5 μm) from the transverse surface(35). The photomicrographs were captured using a computer-facilitated (Motic microscope) system, and for quantification, Image J software was used.

### Special histopathological analysis (PAS and MT staining)

PAS staining was used to identify the deposition of glycogen molecules. For PAS staining, thin sections were cut from paraffin-embedded blocks. The staining was performed as per the reported literature (36) and photographed using a Motic microscope. In the same way, MT staining was also done and photomicrographed, and for quantification, Image J software was used (37). 

### Analysis of NF–ĸB and TGF-β by immunohistochemistry

Thin slices of paraffinized blocks were cut for the immunohistochemical examination. Antigen and microwave retrieval were done after deparaffinization, cleaning, and rehydration of the sample. The slide preparations for immunohistochemical examination of kidney tissues were performed and photomicrographed using a computer-facilitated (Motic microscope) system, and for quantification, Image J software was used (28). 

### Statistical analysis

Values are represented as mean ± SEM. The analysis was done using Graph Pad software (version 8), USA, and applying Tuckey’s multiple comparison tests along with one-way analysis of variance. *P*<0.05 was considered a significant value.

## Results

### Effect of verbenone over CP-mediated oxidative stress in kidney

Kidneys of CP-intoxicated mice showed declined CAT, SOD, GSH, and elevated TBARS levels when compared with the control group (*P*<0.001). VRB 200, VRB 300, and FF 80 treatments greatly enhanced CAT (*P*<0.01, *P*<0.001, and *P*<0.001, respectively), SOD (*P*<0.001, *P*<0.001 and *P*<0.001, respectively), and GSH (*P*<0.01, *P*<0.001, and *P*<0.001, respectively) and significantly reduced MDA levels (*P*<0.001, *P*<0.001 and *P*<0.001, respectively) with regard to CP treated mice (Figure 2). Compared to the control group, the VRB 300 *per se* group did not exhibit any considerable changes in these parameters. 

### Effect of verbenone on CP-mediated kidney injury markers

Compared with the control group, CP-intoxicated mice showed enhanced serum levels of urea, uric acid, BUN, and creatinine (*P*<0.001) in kidney tissues. Treatment with VRB 200, VRB 300, and FF 80 significantly decreased Urea (*P*<0.05, *P*<0.001, and *P*<0.001, respectively), Uric acid (*P*<0.01, *P*<0.001, and *P*<0.001, respectively), BUN (*P*<0.05, *P*<0.001, and *P*<0.001, respectively), and creatinine (*P*<0.05, *P*<0.001, and *P*<0.001, respectively) as compared to the CP treated mice (Figure 3). VRB 300 *per se *group did not show any significant changes in these parameters when compared with the control group. 

### Effect of verbenone on CP-mediated inflammatory markers in the kidney

CP-intoxicated mice showed a significant increase in TNF-α, IL-6, and IL-1β levels and a reduction in IL-10 levels compared to the control (*P*<0.001). Treatment with VRB 200, VRB 300, and FF 80 significantly reduced TNF-α (*P*<0.01, *P*<0.001, and *P*<0.001, respectively), IL-6 (*P*<0.001, *P*<0.001, and *P*<0.001, respectively), IL-1β (*P*<0.001, *P*<0.001, and *P*<0.001, respectively) and increased IL-10 (*P*<0.01, *P*<0.001, and *P*<0.001, respectively) as compared to the CP treated mice (Figure 4). VRB 300 *per se* group did not show any significant changes in these parameters when compared with the control group. 

### Effect of verbenone on CP-mediated histopathology of renal tissue (H&E staining)

CP 200 treated group revealed notable glomerular damage (black arrow), impaired basement membrane (orange arrow), increased pyknosis, cellular disintegration (red arrow), and damaged PCT (Proximal convoluted tubule) and DCT (Distal convoluted tubule), (blue arrow) when compared to the control group which showed normal cellular architecture. When the animals were treated with VRB 200, VRB 300, and FF 80, VRB 300 and FF 80 showed a marked reduction in histopathological aberrations in the renal tissue, whereas VRB 200 did not show any significant recovery. The histomorphology of the *per se* groups was as normal as the control group (Figure 5). 

### Effect of verbenone on CP-mediated renal fibrosis (MT staining)

The CP 200-treated group showed considerably increased collagen deposition in the glomerulus, PCT, and DCT, which signified clear renal fibrosis (black arrow). When treated with VRB 200, VRB 300, and FF 80, there was no significant reduction in the VRB 200 group, whereas the VRB 300 and FF 80 group showed a marked reduction in the deposition of collagen in the glomerulus, PCT, and DCT. VRB 300 *per se *group showed no significant changes in these parameters compared to the control group (Figure 6). 

### Effect of verbenone on CP-mediated glycogen deposition in kidneys (PAS staining)

The CP 200-treated group showed significantly increased glycogen deposition (black arrow). When the animals were treated with VRB 200, VRB 300, and FF 80, VRB 200 showed no significant reduction. However, VRB 300 and FF 80 showed a marked reduction in glycogen deposition in the glomerulus, PCT, and DCT. VRB 300 *per se* group showed no significant changes in these parameters compared to the control group (Figure 7). 

### Immunohistochemistry (IHC) of p-NF–ĸB 

CP 200 treated group revealed significantly increased NF-kB expression (red arrow), which was reduced in VRB 300 and FF 80 groups. VRB 200, however, did not show any significant reduction in the expression. In the control and *per se* groups, there was a minimal expression (black arrow), as shown in Figure 8. 

### Immunohistochemistry (IHC) of TGF-β1

CP 200 treated group revealed significantly increased TGF-β1 expression (red arrow). When the animals were treated with VRB 200, VRB 300, and FF 80, there was no significant reduction in the expression level with VRB 200, whereas VRB 300 and FF 80 manifested considerable reduction in TGF-β1 expression. In the control and *per se* groups, there was a minimal expression (black arrow), as shown in Figure 9. 

## Discussion

In recent years, clinical and preclinical studies have examined the detrimental effects of CP on kidneys (1). However, the precise mechanism by which this occurs remains uncertain. CP-induced oxidative damage plays a pivotal role in causing nephrotoxicity (28). This drug has been reported to directly affect the kidney, causing adverse effects such as degeneration in glomeruli and necrosis of proximal convoluted tubules. It also damages distal tubules and causes pyknosis and other adverse changes (38). Previous literature has revealed that CP administration can cause both acute and chronic renal injuries (39, 40). Moreover, no adjuvant treatments that can reduce the damaging properties of CP are available so far (5). Thinking along this line, we, therefore, tried to investigate the protective role of VRB in CP-induced kidney damage in mice. We have assessed various biochemical markers and histopathological studies (H&E, MT, and PAS staining) to investigate the efficacy of VRB in countering renal toxicity mediated by CP. This study represents the first instance of examining verbenone’s effect on kidney damage mediated via CP. The study successfully exhibited the protective potential of VRB.

The anti-oxidant molecules and enzymes present in the kidney, such as SOD, CAT, and GSH, defend oxidative stress, which ROS and RNS may generate in response to CP metabolism. CP affects various organs differently, but the kidney is particularly vulnerable due to its checkpoint functionality (41). GSH, CAT, and SOD are the body’s endogenous anti-oxidant defense that neutralizes ROS and RNS naturally, but their continuous increase leads to oxidative stress and triggers inflammation and apoptosis. Therefore, a healthy kidney system necessitates carefully balancing ROS, RNS, and endogenous enzymes (42). Lipid peroxidation (MDA) is one of the manifestations of oxidative stress, and its measurement considerably describes the damage done to the organ. Researchers have shown that administration of CP increases lipid peroxidation and renal damage, which can be reversed using natural products rich in anti-oxidant properties (28, 43, 44). In our research, we also observed that when 200 mg/kg of CP was given to mice, the levels of anti-oxidant enzymes were significantly reduced, and MDA was increased. Treatment with VRB 200, VRB 300, and FF 80 considerably reversed the effect to normal, indicating their significant renal protective potential (Figure 2). 

Another way of assessing renal toxicity is by measuring inflammation. Inflammation is often evaluated by measuring the concentrations of several inflammatory indicators, including cytokines (45). The rise in renal cytokines and apoptosis is known to be critically dependent on the generation of reactive oxygen species (46). TNF-α, IL-6, IL-1B (pro-inflammatory), and IL-10 (anti-inflammatory) are the cytokines that play essential roles in the event of inflammation. People have shown that in case of toxicity, pro-inflammatory cytokines increase and anti-inflammatory cytokines decrease (28, 47). CP induces oxidative stress and inflammation, a well-documented phenomenon in preclinical and clinical research (48). NF-κB is a classic indicator of inflammation. Reactive oxygen species, produced during oxidative stress, cause NF-κB activation through IKK/IkBα proteins, leading to inflammation. NF-kB, a transcription factor, migrates into the nucleus and transcribes TNF-α, IL-6, and IL-1β, besides other inflammatory molecules (49, 50). These inflammatory molecules then cause inflammation. Thus, assessing these cytokines gives a sufficient idea of the status of inflammation in the tissue. Our study found that CP causes inflammation in the kidneys, NF-κB signaling modification, and a rise in oxidative strain (40). In our studies, we found that CP administration resulted in the elevation of NF-kB, IL-6, IL-1β, and TNF-α expression and a decline in IL-10, an anti-inflammatory molecule, thereby showing renal inflammation and damage (36, 51). VRB at 300 and FF at 80 mg/kg doses reversed these changes to normal and showed. 

Assessment of kidney-specific indicators such as blood urea nitrogen, serum creatinine, uric acid, and serum urea are other crucial biochemical parameters for determining a drug’s toxicity and assessing the kidney’s well-being (5). These parameters increase when kidney function is compromised. The normal levels of uric acid, urea, blood urea nitrogen, and creatinine indicate normal kidney function and glomerular filtration rate (GFR), whereas the increased levels indicate compromised renal function, reduced glomerular filtration rate (GFR), and nephrotoxic effects (52–54). Our study showed that uric acid, urea, BUN, and serum creatinine levels were increased significantly on CP 200 administration, which decreased to normal with VRB and FF treatment. These findings demonstrated the nephroprotective effect of VRB, as shown in Figure 3. 

Histopathological investigation (H&E staining) further revealed that the glomeruli of the mice were degenerated, the basement membrane was distorted, proximal and distal convoluted tubules were damaged, and pyknosis occurred in the mesangial and endothelial cells, with cellular degeneration on CP administration. Our study was in accordance with the previous studies, which demonstrated increased histopathological abnormalities upon CP exposure (28, 55, 56). VRB 200 and VRB 300 treatments significantly restored renal histopathological abnormalities to normal VRB 300; however, they showed better results than VRB 200. We also tried to determine the effect of CP 200 on glycogen storage in the kidneys and the related complications caused by it. Using PAS stain, we found that the basement membrane of the CP treated group got thickened, showing toxicity. With VRB 300 as an adjuvant, we saw a notable return of these signs to normal, as shown in Figure 6. Our results agree with the earlier reports, which demonstrated that CP 200 caused the thickening of mesenchymal and glomerular basement membranes, as glycogen was deposited in both areas (36, 38, 55, 57). Fibrosis is another manifestation of toxicity seen with CP use (58). Our studies also tried to measure fibrosis by estimating TGF-β1 and histopathologically by MT staining. The mechanism of TGF-β1 is that it triggers the stimulation and transition of various cell types in the kidney, leading to the formation of fibrogenic cells, including myofibroblasts and interstitial fibroblasts (59). We found increased TGF-β1 expression and collagen deposition in the kidneys’ glomerular and proximal/distal convoluted tubules. This condition was significantly reversed with the use of VRB and FF. All the parameters and assessments in this study clearly indicate that verbenone is a potential molecule that can protect kidneys and reverse the adverse effect of CP in mice. 

**Table 1:** Plan of treatment among different groups of Swiss albino mice

S. No.	Group (n = 6)	Dosage, method, and period
1	Vehicle control	0.5 ml corn oil orally for 14 days.
2	CP (Toxic)	200 mg/kg CP, single shot intraperitoneally on the seventh day
3	VRB_200 _+ CP (Treatment 1)	200 mg/kg, p.o. VRB (for 14 days) + 200 mg/kg CP, single shot intraperitoneally on the seventh day
4	VRB_300 _+ CP (Treatment 2)	300 mg/kg, p.o. VRB (for 14 days) + 200 mg/kg CP, single shot intraperitoneally on the seventh day
5	FF_80 _+ CP (Standard)	80 mg/kg, p.o. FF (for 14 days) + 200 mg/kg CP, single shot intraperitoneally on the seventh day
6	VRB_300_ *per se* ( *per se* group)	300 mg/kg, p.o. VRB (for 14 days)

CP: Cyclophosphamide, VRB: Verbenone, FF: Fenofibrate

**Figure 1:**
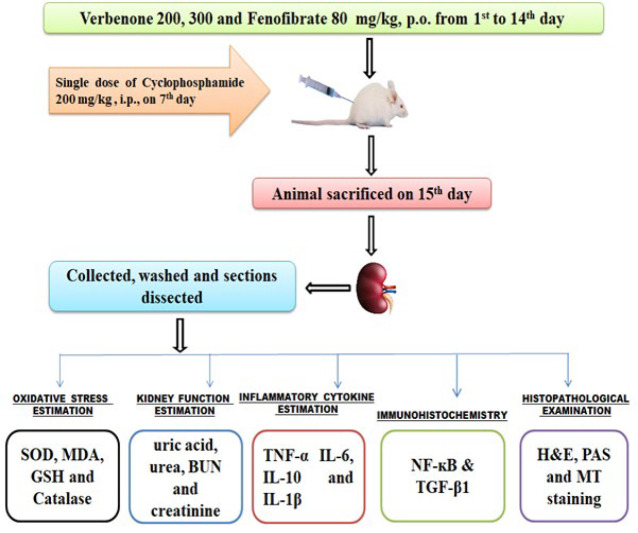
Overall map of the treatment protocol in Swiss albino mice

Various oxidative stress parameters (SOD: superoxide dismutase; CAT: catalase; GSH: glutathione; MDA: malondialdehyde), Kidney function parameters (BUN: blood urea nitrogen; UA: uric acid; Cr: creatinine; serum urea); Inflammatory parameters (TNF-α, tumor necrosis factor α; ILs, interleukins; TGF-β, transforming growth factor beta; NF-κB, nuclear factor kappa-light-chain enhancer of activated B cells; and Histopathological changes (H&E, hematoxylin and eosin; PAS, periodic acid–Schiff; and MT, Masson's trichrome staining) were assessed to ascertain the nephrotoxicity caused by CP (cyclophosphamide) and nephroprotection produced by VRB (verbenone) and FF (fenofibrate).

**Figure 2:**
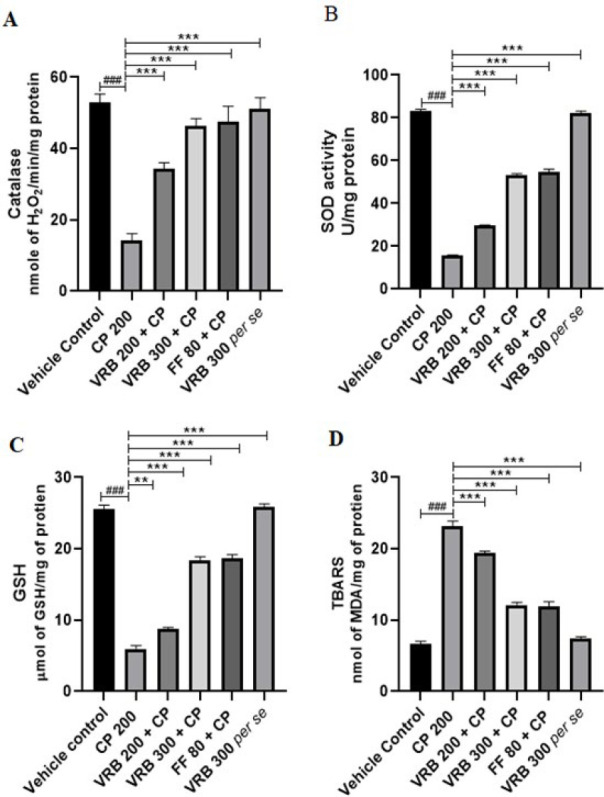
Effect of CP (cyclophosphamide), VRB (verbenone) 200, 300 and FF (fenofibrate) 80 on oxidative stress markers (CAT, SOD, GSH and MDA) in renal tissue of Swiss albino mice

CP-intoxicated mice remarkably decreased CAT, SOD and GSH levels and increased MDA levels in the kidney tissue. Pretreatment with VRB 200, VRB 300, along with FF 80 significantly reversed CAT, SOD, GSH, and MDA towards normal. The *per se *group did not show any significant changes. The statistical data is shown as mean ± SEM (n = 6). To assess the statistical significance of the data, one-way analysis of variance (ANOVA) was employed and then succeeded by Tuckey’s Honest Significant Difference test, ns is non-significant vs CP, and ***P<0.001 significant, vs control; #*P*<0.05, ##*P*<0.01, ###*P*<0.001 significant vs CP.

**Figure 3:**
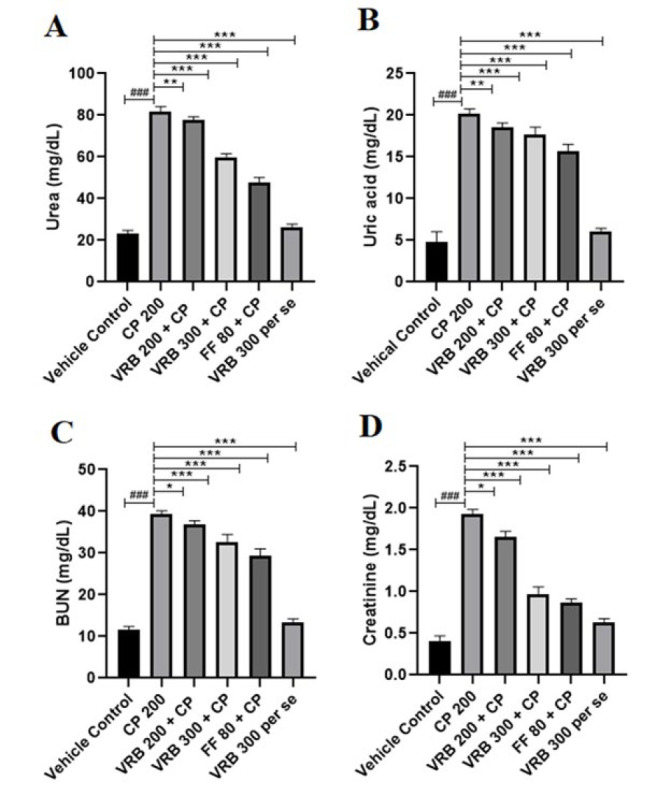
Effect of CP (cyclophosphamide), VRB (verbenone) 200, 300 and FF (fenofibrate) 80 on renal injury markers (Urea; UA, uric acid; BUN, blood urea nitrogen; and Cr, creatinine) of Swiss albino mice

CP-intoxicated mice remarkably increased the kidney tissue's Urea, UA, BUN, and Cr levels. Pretreatment with VRB 200, VRB 300, and FF 80 significantly reversed Urea, UA, BUN, and Cr towards normal. The *per se *group showed no significant changes compared to the control group. To assess the statistical significance of the data, one-way analysis of variance (ANOVA) was employed and then succeeded by Tuckey’s Honest Significant Difference test, ns is non-significant vs CP, and ***P<0.001 significant, vs control; #*P*<0.05, ##*P*<0.01, ###*P*<0.001 significant vs CP.

**Figure 4:**
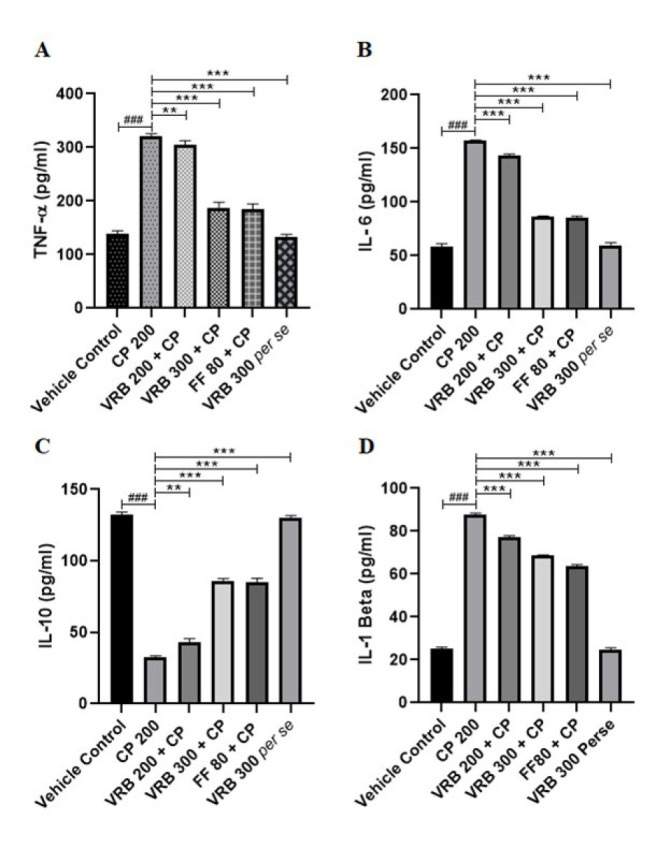
Effect of CP, VRB 200, VRB 300 and FF 80 on inflammatory markers (IL-6, IL-10, IL-1β &TNF-α) of renal tissue in Swiss albino mice

Mice intoxicated with CP displayed marked increases in IL-6, IL-1β, and TNF-α levels in their kidney tissue, while IL-10 levels were reduced. Pretreatment with VRB 200, VRB 300, and FF 80 significantly reversed IL-6, IL-10, IL-1β, and TNF-α toward normal. The *per se *group did not show any significant changes. To assess the statistical significance of the data, one-way analysis of variance (ANOVA) was employed and then succeeded by Tuckey’s Honest Significant Difference test, ns is non-significant vs CP, and ***P<0.001 significant, vs control; #*P*<0.05, ##*P*<0.01, ###*P*<0.001 significant vs CP.

**Figure 5:**
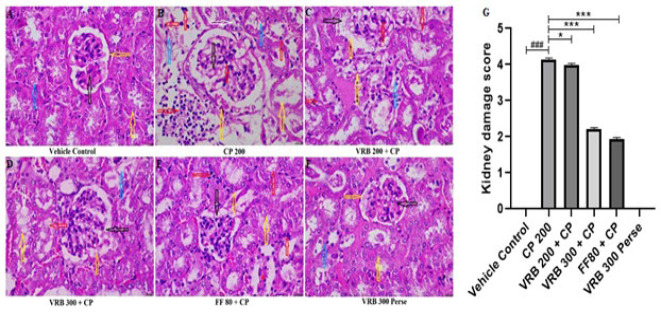
Images from (A-F) show the histological changes (H&E staining, scale bar 50 µm) and Figure G represents the semi-quantitative analysis of kidney injury score of renal tissue in various groups of Swiss albino mice

[A] Vehicle control showing normal cellular architecture, [B] CP 200 (Toxic group) showing notable glomerular damage (black arrow), impaired basement membrane (orange arrow), increased pyknosis and cellular disintegration (red arrow), and damaged proximal/distal convoluted tubules (blue arrow), when compared to the control group. [C] The VRB 200 (Treatment 1) group showed a non-significant toxicity reversal compared to the toxic group. [D] VRB 300 (Treatment 2) group showed a significant decrease in pyknosis, glomerular and tubular damage, basement membrane impairment, and cellular disintegration compared to the toxic group. [E] FF 80 (Standard) group also showed a significant decrease in pyknosis, glomerular and tubular damage, basement membrane impairment, and cellular disintegration compared to the toxic group. [F] VRB 300 *per se *group showing cellular architecture similar to the vehicle control in terms of no pyknosis, glomerular and tubular damage, basement membrane impairment, and cellular disintegration.

**Figure 6:**
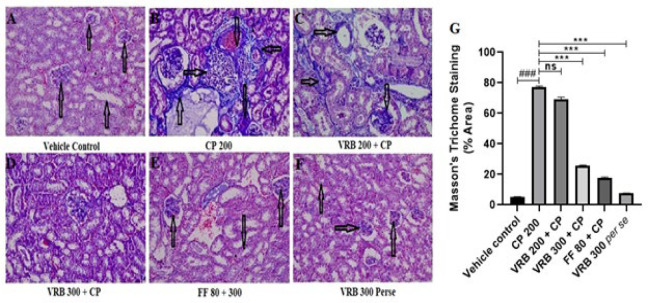
Images from (A-F) show the Masson’s Trichome (MT) staining (MT, scale bar 50 µm) and Figure G represents the semi-quantitative analysis of MT staining of renal tissue in various groups of Swiss albino mice

[A] Vehicle control showing no collagen deposition in the glomerulus, PCT/DCT, [B] CP 200 (Toxic group) showing considerably increased collagen deposition in the glomerulus, PCT/DCT and hence signified clear renal fibrosis (black arrow), when compared to the control group. [C] VRB 200 (Treatment 1) group showed a non-significant reduction of collagen deposition in the glomerulus, PCT/DCT compared to the toxic group. [D] VRB 300 (Treatment 2) group showed a marked reduction in collagen deposition in the glomerulus, PCT/DCT compared to the toxic group. [E] The FF 80 (Standard) group also showed a marked reduction in collagen deposition in the glomerulus and PCT/DCT compared to the toxic group. [F] VRB 300 *per se *group showing cellular architecture similar to the vehicle control regarding collagen deposition in the glomerulus, PCT/DCT.

**Figure 7:**
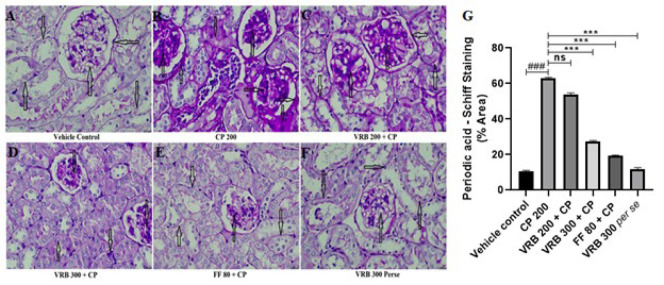
Images from (A-F) show the Periodic Acid-Schiff (PAS) staining (PAS, scale bar 50 µm) and Figure G represents the semi-quantitative analysis of PAS staining in various groups of Swiss albino mice

[A] Vehicle control group showing no glycogen deposition in the glomerulus, PCT/DCT, [B] CP 200 (Toxic group) showing considerably increased deposition of glycogen in the glomerulus, PCT/DCT (black arrow), when compared to the control group. [C] VRB 200 (Treatment 1) group showing non-significant reduction of glycogen deposition in the glomerulus, PCT/DCT when compared with the toxic group. [D] The VRB 300 (Treatment 2) group showed a marked reduction in the glycogen deposition in the glomerulus, PCT/DCT, compared to the toxic group. [E] The FF 80 (Standard) group also showed a marked reduction in glycogen deposition in the glomerulus and PCT/DCT compared to the toxic group. [F] VRB 300 *per se *group showing cellular architecture similar to the vehicle control in terms of glycogen deposition in the glomerulus, PCT/DCT.

**Figure 8:**
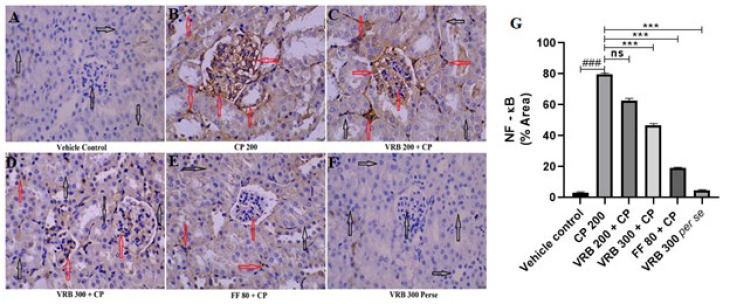
Images from (A-F) showing the impact of CP on renal tissue’s NF-κB expression via immunohistochemistry staining (IHC, NF-kB, scale bar 50 μm) and Figure G represents the semi-quantitative analysis of NF-κB level in various groups of Swiss albino mice

[A] Vehicle control group showing minimal expression in NF-kB level (black arrow), [B] CP 200 (Toxic group) showing significant increase in NF-kB expression (red arrow) when compared to the control group. [C] The VRB 200 (Treatment 1) group showed a non-significant reduction of NF-kB expression compared to the toxic group. [D] The VRB 300 (Treatment 2) group showed a significant reduction of NF-kB expression compared to the toxic group. [E] The FF 80 (Standard) group also showed a significant reduction in NF-kB

expression compared with the toxic group. [F] VRB 300 *per se *group showing similar NF-kB expression as the vehicle control (black arrow).

**Figure 9:**
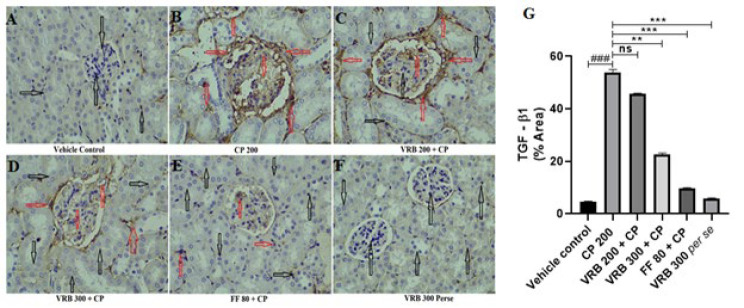
Images from (A-F) showing the impact of CP on renal tissue’s TGF-β1 expression via immunohistochemistry staining (IHC, TGF-β, scale bar 50 μm) and Figure G represents the semi-quantitative analysis of TGF-β1 in various groups of Swiss albino mice

[A] Vehicle control group showed minimal expression in TGF-β1 level (black arrow), [B] CP 200 (Toxic group) showed a significant increase in TGF-β1 expression (red arrow) when compared to the control group. [C] The VRB 200 (Treatment 1) group showed a non-significant reduction of TGF-β1 expression compared to the toxic group. [D] VRB 300 (Treatment 2) group showed a significant reduction of TGF-β1 expression compared to the toxic group. [E] FF 80 (Standard) group also showed a significant reduction of TGF-β1

expression compared to the toxic group. [F] VRB 300 *per se *group showing similar TGF-β1 expression as the vehicle control (black arrow).

## Conclusion

Verbenone significantly plays a role in protection against cyclophosphamide-induced renal damage. This protection was shown by both the concentration of the drug, i.e., 200 and 300 mg/kg body weight; however, 300 mg/kg body weight was found more potent in offering renal protection. Verbenone, being a natural product with fewer adverse effects, may become a better choice for cancer patients who are undergoing chemotherapy and have a chance of developing renal toxicity as a side effect of CP. 

## Declaration

We have not used any AI tools or technologies to prepare this manuscript.
